# Effect of Purine Nucleoside Analogue-Acyclovir on The Sperm Parameters and Testosterone Production in Rats

**Published:** 2013-03-06

**Authors:** Elham Movahed, Rajabali Sadrkhanlou, Abbas Ahmadi, Vahid Nejati, Zahra Zamani

**Affiliations:** 1 Department of Biology, Faculty of Science, Urmia University, Urmia, Iran; 2 Laboratory of Embryology, Department of Basic Science, Faculty of Veterinary Medicine, Urmia University, Urmia, Iran

**Keywords:** Acyclovir, Sperm Parameters, Testosterone, Rat, Antiviral Drugs

## Abstract

**Background:**

Acyclovir (ACV), a synthetic purine nucleoside analogue derived from guanosine, is known to be toxic to gonads and the aim of this study was to evaluate the effect of ACV on the sperm parameters and testosterone production in rat.

**Materials and Methods:**

In this experimental study, forty adult male Wistar rats (220 ± 20 g) were randomly divided into five groups (n=8 for each group). One group served as control and one group served as sham control [distilled water was intraperitoneally (i.p.) injected]. ACV was administered intraperitoneally in the drug treatment groups (4, 16 and 48 mg/kg/day) for 15 days. Eighteen days after the last injection, rats were sacrificed by CO_2_ inhalation. After that, cauda epididymides were removed surgically. At the end, sperm concentrations in the cauda epididymis, sperm motility, morphology, viability, chromatin quality and DNA integrity were analyzed. Serum testosterone concentrations were determined.

**Results:**

The results showed that ACV did not affect sperm count, but decreased sperm motility and sperm viability at 16 and 48 mg/kg dose-levels. Sperm abnormalities increased at 48 mg/kg dose-level of ACV. Further, ACV significantly increases DNA damage at 16 and 48 mg/kg dose-levels and chromatin abnormality at all doses. Besides, a significant decrease in serum testosterone concentrations was observed at 16 and 48 mg/ kg doses.

**Conclusion:**

The present results highly support the idea that ACV induces testicular toxicity by adverse effects on the sperm parameters and serum level of testosterone in male rats.

## Introduction

Antiviral drugs are often nucleoside analogues that are known to have potential teratogenic, embryotoxic, carcinogenic and antiproliferative activities (1). The reproductive system is very sensitive to toxic chemicals because of the high multiplication rate of germ cells that result in high susceptibility of male gonad to toxic effect of chemotherapy (2, 3). On the other hand this is the only system in which genetic damage from one generation to another can transfer. Thus, it is important to investigate the cytotoxic and genotoxic effects of various agents on germinal cells (1). Apparently the spermato-toxic effects of antiviral drugs are not well studied. An antiviral drug-ribavirin, which is aninosinemonophosphat dehydrogenase inhibitor, has been reported to induce cytotoxicity and genotoxicity damages in germ cells and also to the general structure of the testis (4). Previous studies have shownthat another antiviral drug, Gancyclovir, induces testicular damage and germ cell apoptosis in transgenic mouse (5). Also, interferon alpha-2 has been demonstrated to causes wide spread structural changes in rat testis (6).

ACV [9-(2-hydroxyethoxymethyl) guanine] is a synthetic purine nucleoside analogue that was introduced as the fifth anivirals drug commonly used in the early 1980’s. ACV has been reported to be very effective against the treatment of herpes simplex and varicella zoster infections and it also protects immune-suppressed patients that receive transplants from cytomegalovirus (7, 8). ACV inhibits viral DNA replication effectively much more than cellular DNA replication indicating that ACV can mildly impair host cells. ACV is nonmutagenic in Ames test, a biological assay to assess the mutagenic potential of chemical compounds. However, cell division of HeLa cells exposed to ACV was completely inhibited but the cell number did not change significantly. Also ACV has been reported to induce the formation of micronuclei indicating its ability to destroy the chromosome structure (9).

The androgen production and spermatogenesis are two main testicular functions. The interstitial Leydig cells produce testosterone which is a kind of androgen. Spermatogenesis occurs in seminiferous tubules. Gonadotropins affect normal spermatogenesis qualitatively and quantitatively (10). Testosterone receptors are located on sertoli and peritubularmyoid cells. Thus, testosterone indirectly affects spermatogenesis by binding to its receptors (11).

Spermatogonia are sensitive to toxins interfering with DNA replication because these cells go through several mitotic divisions (12). Considering the complexity of spermatozoan functions in fertilization, measuring multiple sperm parameters than comparing any single parameter provides a more complete estimate of sperm quality (13). Thus in this study, several sperm parameters and also the serum level of testosterone in male rats were evaluated.

## Materials and Methods

### Animals

In this study forty male Wistar rats (220 ± 20 g) were obtained from animal house of Faculty of Science, Urmia University and kept under specific conditions on a constant 12-hour light/dark cycle and at a controlled temperature of 22 ± 2˚C. Standard pellet food and tap water were available ad libitum. Animals were allowed to acclimatise for one week before experimental use. It should be noted that this study was an experimental study accordance with the Guidance of Ethical Committee for research on Laboratory Animals of Urmia University.

### Drugs

ACV (MYLAN, France) was used at three dose levels, 4, 16 and 48 mg/kg based on previous studies (1, 9). Drug was dissolved in distilled water before injection.

### Drug treatment

Animals were segregated into 5 groups of eight each. Group 1 served as control, normal and apparently healthy rats that did not receive any type of treatment. Group 2 served as sham control and received distilled water (i.p. injection) for 15 consecutive days. Groups 3, 4 and 5 (the drug treated groups) were administered respectively 4, 16 and 48 mg/kg/day ACV (i.p. injection) for 15 consecutive days.

18 days after the last injection 4 animals from each group were sacrificed by CO_2_ inhalation. The blood samples were collected from jugular vein andsubsequently the serumwas harvested and frozen. The testes were removed surgically. Total experimental duration was 33 days.

### Sperm collection

Left epididymal sperms were collected by slicing the epididymides in 5 ml of human tubal fluid (HTF) +4 mg/ml bovine serum albumin (BSA) and incubating for 5 min at 37˚C in an atmosphere of 5% CO_2_ to allow sperm to swim out of the epididymal tubules.

### Sperm count

The epididymal sperm count was determined by hemocytometry (Neubauer chamber) and the method described in the WHO manual (1999) (14). A 5μl aliquot of epididymal sperm was diluted with 95μl of diluent (0.35% formalin containing 5% NaHCO_3_ and 0.25% trypan blue). A few drops of the diluted sperm suspension as a sample, was transferred into a Neubauer’s improved counting chamber (depth 0.1 mm), and allowed to stand for 5 minutes. The sperm heads were counted and expressed as million/ml of suspension.

### Sperm morphology

A part of sperm suspension was used for preparing smears to evaluate the sperm shape abnormalities (15, 16). The sperm morphology was also determined using Eosin/Nigrosin stain. To test, one drop of 1% eosin Y and nigrosin was added to the suspension and were mixed by gentleagitiation. Next, smears were prepared on clean and grease-free glass slides, and allowed to dry in air overnight. Preferably, 400 sperms were examined per animal morphologically at 400 magnification. Morphological abnormalities were classified as amorphous head, hookless, banana and doubleheaded, coiled with microcephaly, bent at cephalocaudal junction, bent with projecting filaments, microcephaly with tail defect and defective head with duplication of tail (17).

### Sperm viability

Sperm viability was evaluated as follows. A 20 μl of 0.5% eosin Y and nigrosin were added into an equal volume of the sperm suspension. After 2 min of incubation at room temperature, slides were viewed by light microscope with magnification of 400. Dead sperms appeared to be pink and live sperms were not stained. In each sample 400 sperms were counted and viability percentages were calculated (16).

### Sperm motility

The spermatozoa were divided as motile or immotile. Motility of the spermatozoa was evaluated under a light microscope (Olympus Co., Tokyo, Japan). One drop of sperm suspension was placed on a glass slide, covered with a coverslip, and 10 random fields of view were examined at 400× magnification. The number of motile and nonmotile sperm was counted. Motility was then expressed as the percentage of motile sperm to the total number of sperm (14).

### Acridine-orange DNA denaturation assa

Male infertility and abnormal spermatogenesis have close relation with sperm DNA damage (18, 19). Altered chromatin structure measured by susceptibility of sperm DNA to acid-induced denaturation was assessed with acridine-orange (AO). AO intercalates into native DNA and the dye fluoresces green when exposed to blue light and red light when bound to single- stranded DNA. Thick smears were placed in Carnoy’s fixative (methanol: acetic acid 1:3) for 2 hours forfixation. After staining for 5 min the slides were rinsed with deionized water. Under the fluorescent light microscope, red and green sperms could be observed. For each staining protocol four-hundred sperms were evaluated. Green sperms were classified as normal DNA and yellow to red sperms were classified as damaged DNA (19).

### Aniline blue chromatin quality assay

Histones are replaced by transition proteins and then by protamines during the later stages of spermatogenesis, spermatid nuclear changing and condensing. The DNA strands are tightly wrapped around the protamine molecules to create toroidal structures (19). Protamine-rich nuclei of mature spermatozoa are rich in arginine and cysteine and contain relatively low levels of lysine, therefore they could not be stained by aniline blue (AB). Slides were prepared by smearing 5 μl of either a raw or washed semen sample. The slides are air-dried and fixed for 30 minutes in 3% glutaraldehyde in phosphate buffered saline. The smear was dried and stained for 5 minutes in 5% aqueous aniline blue solution (pH=3.5). Sperm heads containing immature nuclear chromatin stain blue and those with mature nuclei do not take up the stain. The percentage of spermatozoa stained with aniline blue was determined by counting 400 spermatozoa per slide under bright field microscope (20).

### Electrochemiluminescence (ECL)

After blood sampling the serum was separated using a centrifuge and kept at -70˚C until analysis of testosterone hormone. Serum testosterone concentrations were measured by using a testosterone Electrochemiluminescence Kit (Roche Diagnostics, Germany, Limit of Detection: 0.025 ng/ml).

### Statistical analysis

The data are presented as the mean ± SEM. Differences between groups were analyzed by One Way Analysis of Variance (ANOVA) followed by Tukey test using SPSS package, version 16 and level of significance was taken as p<0.05.

## Results

### Sperm parameters

**Fig 1:**
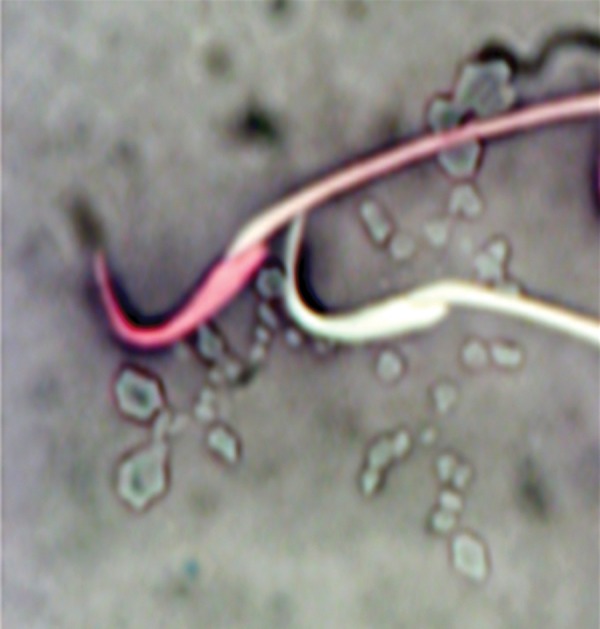
Dead sperm appear pink and live sperm are not stained, Eosin/Nigrosinstaining technique (×2000).

**Fig 2:**
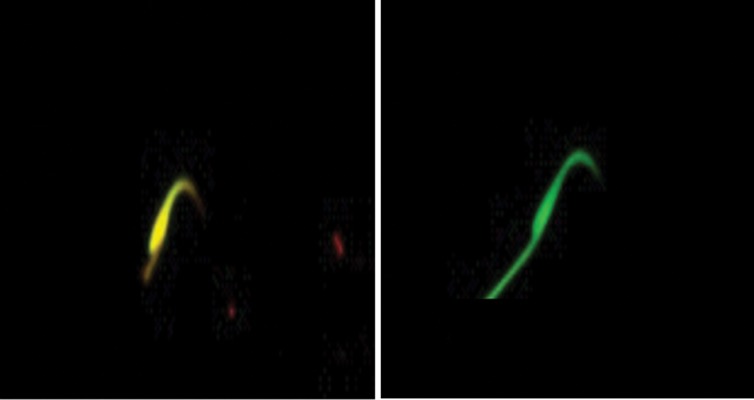
A. Sperm with damaged DNA (yellow), B. Sperm with normal DNA (green). Acridine-orange stainingtechnique (×2000).

**Fig 3:**
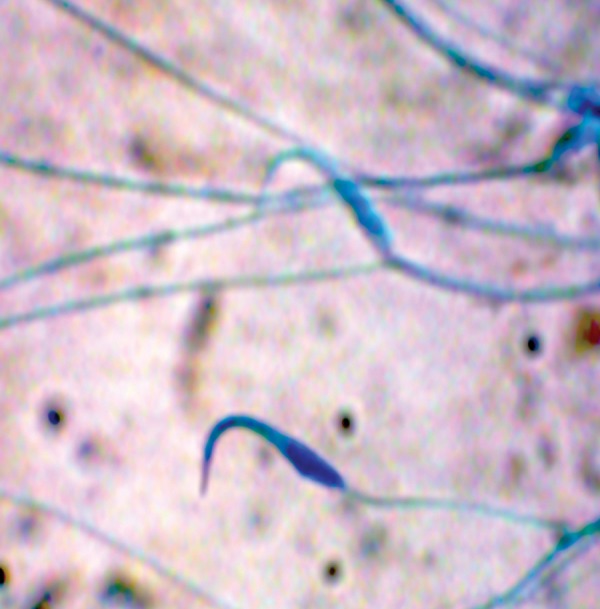
Sperm head containing immature nuclear chromatin is dark blue and sperm head with mature nuclei is light blue. Aniline blue staining technique (×2000).

Result showed that i.p. injection of ACV did not cause significant changes in total cauda epididymal sperm count as compared to control and sham control groups (Table 1). Treatment with ACV caused significant decrease in sperm motility at 16 and 48mg/kg dose-levels (p<0.01) in dose dependent manner (Table 1). The percentage of sperm morphological abnormalities significantly increased in ACV treated rats only at 48 mg/kg dose-level (p<0.05) in a dose dependent manner Movahed et al. (Table 1). The percentage of live spermatozoa in animals exposed to ACV at doses of 16 mg/ kg (p<0.05) and 48 mg/kg (p<0.01) were significantly lower than control and sham control groups in dose dependent manner (Table 1, Fig 1). The percentage of spermatozoa with DNA damage significantly increased at 16 and 48 mg/kg dose- levels (p<0.01) in a dose dependent manner (Table 2, Fig 2). Treatment with ACV at all dose-levels significantly increased the percentage of spermatozoa with chromatin abnormalities (p<0.01) in dose dependent manner (Table 2, Fig 3).

### Serum testosterone level

**Fig 4:**
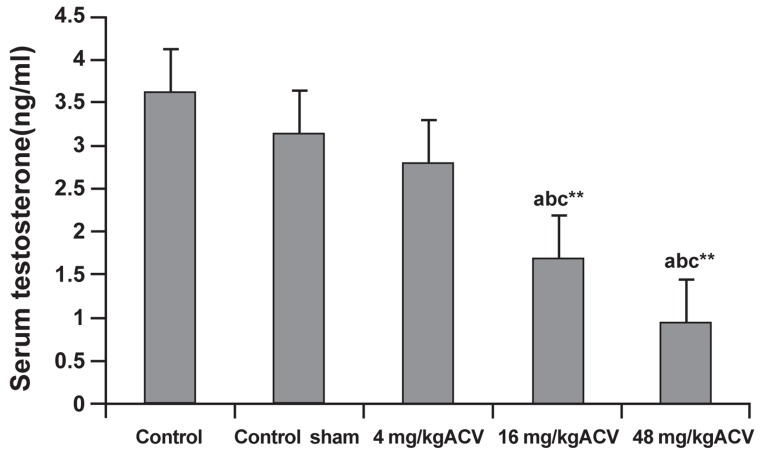
Effects of ACV on serum testosterone concentrations in adult male rats. 4 rats from each group were analysedin this experiment. Error bars indicate the standard error of the mean. *; p<0.05 and **; p<0.01. a; Significant compared with control, b; Significant compared with sham control and c; Significant compared with 4 mg/kg ACV.

ACV significantly decreased serum testosterone level at 16 and 48 mg/kg dose-levels (p<0.01) in a dose dependent manner (Fig 4).

**Table 1:** Effects of ACV on sperm parameters in adult male rats


	Total sperm/cauda epididymis (10^6^)	Motile sperm (%)	Abnormal sperm (%)	Live sperm (%)

**Control**	205.2 ± 1.314	67.25 ± 2.174	11.5 ± 0.866	69.50 ± 0.866
**Sham control **	205 ± 1.04	67 ± 2.121	11.25 ± 1.25	68.25 ± 1.376
**4 mg/kg ACV **	230 ± 1.354	55.75 ± 2.393	18.25 ± 1.6	59.50 ± 2.179
**16 mg/kg ACV**	185 ± 1.755	41 ± 2.041^ab**c*^	19.5 ± 4.051	58.25 ± 2.25^ab*^
**48 mg/kg ACV**	172.5 ± 1.937	37 ± 6.069^abc**^	24.5 ± 2.901^ab*^	41.25 ± 4.366^abc**^


Data are presented as mean ± SEM from 4 animals per group. a; significant compared with control, b; significant compared with sham control and c; significant compared with 4mg/kg ACV. *; P<0.05 and **; P<0.01.

**Table 2:** Effects of ACV on DNA damage and Chromatin abnormalities of sperm in adult male rats


	AO^+^ (%)	AB^+^ (%)

**Control**	11.5 ± 1.258	10 ± 1.08
**Sham control**	9 ± 1.471	7.5 ± 1.707
**4 mg/kg ACV**	19 ± 0.816	27.55 ± 2.561 ^ab**^
**16 mg/kg ACV **	32.5 ± 2.217 ^ab**c*^	25 ± 2.345 ^ab**^
**48 mg/kg ACV**	37.75 ± 5.498 ^abc**^	35.75 ± 2.675 ^ab**d*^


Data are presented as mean ± SEM from 4 animals per group. AO^+^; Acridine-orange positive, AB^+^; Aniline blue positive, a; significant compared with control , b; significant compared with sham control, c; significant compared with 4 mg/kg ACV, d; significant compared with 16mg/kg ACV, *; P<0.05 and **; P<0.01.

## Discussion

In the current study, it was investigated whether a purine nucleoside analogue-acyclovir has any reproductive toxic effects in adult male rats or not. ACV was administrated to rats at doses of 4, 16 and 48 mg/kg body weight (b.w.) These doses were chosen according to a preparative study which investigated the effect of doses of 4, 16, 32 and 48 mg/kg b.w of ACV on male reproductive system in mice (1, 9). Also in this study one group served as control and one group served as sham control.

ACV is reported to inhibit thymidine kinase in the viral defected DNA and also in non-infected cells (21). ACV at dose of 50-100 μM can also cause inhibition of cell division (21). It has been reported to increase the LDH level in the testis, indicating cytotoxicity and extensive tissue damage induced by ACV (9). ACV showed its efficacy to damage cellular DNA in non-infected cells (22).

Sperm count reduction is an important indicator of male infertility (23). Changes in the germ cell function can alter sperm count. Any agent that interferes with mitotic division is also known to reduce the sperm count (24). Previous studies have shownthat some antiviral drugs such as Ribavirin and Gancyclovir decreases sperm count (25-27). This study did not show any significant change in sperm count in the groups treated with ACV in comparison to controls and sham controls but in other studies ACV reduced sperm count in male mice (1, 9). Considering our treatment was 33 days and that which the length of spermatogenesis in rat is approximately 39-45 days, normal sperm count in all animals treated with ACV can be related to previous cycles of spermatogenesis. On the other hand, the results of present study show that the highest dose of ACV causes significant increase in the percentage of sperms with abnormal shape. Similar to ACV, Ribavirin was reported to cause sperm abnormalities in rats in a dose dependent manner (25). Induced sperm abnormalities indicate point mutations in germ cells, which should have triggered structural changes in cell organelles involved in head and tail formation, leadto sperm abnormalities (28). The increase in sperm abnormalities in this study indicates that ACV induced the DNA damage in germ cells as revealed by sperm DNA assay in the present study, leadingto altered sperm morphology. Also increase in abnormal sperm could be related to decrease in testosterone concentration indicating that depletion of testosterone may have some effect on morphogenesis of sperms. The effect of ACV on sperm morphology observed in this study was also reported by Narayana in which male mice were exposed to ACV (9).

Sperm motility often indicates chemical-induced testicular toxicity (29). Also in men, defect in sperm motility causes untreatable infertility or subinfertility (30). In this study the progressive sperm motility and sperm viability of rats treated with ACV significantly decreased, except for the lowest dose of ACV that indicates cytotoxicity of ACV. Moreover, it has previously been demonstrated that male rats exposed to ACV showed significant decrease in sperm motility (1, 9). Also, reduced sperm motility was observed in male rats treated with another antiviral drug zidovudin (31). The negative effect of ACV on sperm viability could be related to the inhibition of cell viability.

It was evident that different factors can induce DNA damage in male germ cells that can result in adverse effects in offspring (32). Many studies have demonstrated that infertile patients with male factor infertility possess hidden anomalies in the composition of their sperm nuclei, displaying a higher level of loosely chromatin and damaged DNA (28). Spermatozoa with DNA damage can show normal zona pellucida binding characteristics and fertilize the oocyte and produce an earlystage embryo, but they failed to produce a successful full-term pregnancy (33, 34). Abnormal sperm chromatin damage in humans and some animals was associated with abnormal chromatin decondensation patterns and a longer interval to the initiation of pronucleus formation after fertilization (35). In a recent study, changes to the highly defined architecture of sperm chromatin have been demonstrated to affect the initiation and regulation of paternal gene expression in early embryos (33). Another study showed the relation of the content of P1 and P2 protamines with sperm chromatin stability (35). Also presence of DNA damage has been reported to have a close relation with infertility (36).

Furthermore,according to previous studies, to package DNA properly during spermatogenesis, protamines are required (37). On the other hand protamination status of chromatin can protect sperm DNA from external factors and impaired chromatin packing may result in damaged sperm DNA (38). ACV is known to be clastogenic in somatic cells revealed by the formation of micronuclei in cultured HeLa cells (35, 39, 40) and in the polychromatic erythrocytes in mouse bone marrow (41), and chromosomal damage in the human lymphocyte (22). This study also showed that ACV significantly increases DNA damage at 16 and 48 mg/kg dose-levels and chromatin abnormality at all dose-levels in rat sperms treated with ACV indicating genotoxicity of this antiviral drug.

It is well known that in the adult, testosterone supports spermatogenesis, sperm maturation and sexual function. Therefore, disruption of testosterone biosynthesis in Leydig cells can adversely affect male fertility (22). Narayana reported that ACV decreases intratesticular testosterone level in male mice (9). Our study also showed that ACV at doses of 16 and 48 mg/kg significantly decreases serum testosterone level in male rats. Therefore, the results demonstrate that ACV can impair spermatogenesis, sperm maturation and sexual function. It should be mentioned that fertility disorders of ACV in humans and animals were not reported hitherto.

## Conclusion

In conclusion, the present study shows that ACV plays negative roles on the reproductive system and function in sexually mature male rats by its adverse effects on the sperm parameters and testosterone production in these animals. Considering the human germ cells have the same sensitivity as that of rat germ cells, cytotoxicity and gonadotoxicity of ACV in humans can be expected. Whether the adverse effects of ACV on male rat fertility are temporary, it remains for further investigation.
